# Chemokine Receptor 5 Δ32 Allele in Patients with Severe Pandemic (H1N1) 2009

**DOI:** 10.3201/eid1610.100108

**Published:** 2010-10

**Authors:** Yoav Keynan, Jennifer Juno, Adrienne Meyers, T. Blake Ball, Anand Kumar, Ethan Rubinstein, Keith R. Fowke

**Affiliations:** Author affiliations: University of Manitoba, Winnipeg, Manitoba, Canada (Y. Keynan, J. Juno, A. Meyers, T.B. Ball, A. Kumar, E. Rubinstein, K.R. Fowke);; Public Health Agency of Canada, Winnipeg (A. Meyers, T.B. Ball)

**Keywords:** Influenza, CCR5, pandemic (H1N1) 2009, viruses, dispatch

## Abstract

Because chemokine receptor 5 (CCR5) may have a role in pulmonary immune response, we explored whether patients with severe pandemic (H1N1) 2009 were more likely to carry the CCR5Δ32 allele than were members of the general population. We found a large proportion of heterozygosity for the CCR5Δ32 allele among white patients with severe disease.

Chemokine receptor 5 (CCR5) is a protein that belongs to the β-chemokine receptor family and is expressed primarily on T cells, macrophages, and dendritic cells. CCR5 plays a role in mediating leukocyte chemotaxis in response to its ligands, which include RANTES, MIP-1a, and MIP-1b. It may help direct many immune cell subsets, including regulatory T cells and Th17 cells, to sites of infection. CCR5 is also 1 of 2 common co-receptors for HIV. Until recently, understanding the role of CCR5 in supporting the antiviral immune response was limited to appreciation of the role of receptor deficiency in protecting from HIV infection and disease progression. Persons who are homozygous for the CCR5Δ32 allele, a condition in which a 32-bp deletion in the CCR5 gene prevents its expression on the cell surface, have been shown to have reduced susceptibility to HIV infection; the heterozygous state delays HIV disease progression (*1*–*3*). However, homozygosity of the Δ32 allele has recently been shown to be associated with increased risk for symptomatic and fatal West Nile virus infection (*4*). This association was confirmed in a larger meta-analysis (*5*); CCR5 facilitated directed movement of lymphocytes during infection in a mouse model of West Nile virus infection (*6*). A case report of an adverse reaction to the yellow fever virus vaccine in a person heterozygous for CCR5Δ32 and a link between the CCR5Δ32 allele and severe tickborne encephalitis symptoms suggest that CCR5 may play a role in the immune response to other flavivirus infections as well (*7*,*8*). Several reports have suggested a potential effect of the CCR5Δ32 allele on the response to influenza viruses. In mouse models, CCR5 is pivotal in directing CD8+ T cells to lung airways during challenge with Sendai virus (*9*); similarly, deaths among CCR5^–/–^ mice increase after infection with influenza A virus (*10*). Because of the range of severity of recent pandemic (H1N1) 2009 infections and the possible role for CCR5 in the pulmonary immune response, we sought to determine whether patients requiring intensive care admission and respiratory support for severe pandemic (H1N1) 2009 were more likely to carry the CCR5Δ32 allele than were members of the general population.

## The Study

In response to the outbreak of pandemic (H1N1) 2009 in Mexico, we conducted an observational study of critically ill patients with this infection in Winnipeg, Canada. The research was approved by the local research ethics board. The study protocol is described in detail (*11*).

We examined blood samples from 20 patients with laboratory-confirmed pandemic (H1N1) 2009. Average patient age was 40.35 years. Ethnicity was nonwhite for 10 patients, white for 9, and unknown for 1.

Peripheral blood mononuclear cells were stored, and a subset of samples were thawed and resuspended in 200 uL phosphate-buffered saline. Genomic DNA was extracted by using the QIAamp DNA Mini Kit (QIAGEN, Valencia, CA, USA) according to the manufacturer’s instructions. DNA was amplified by using previously reported primers surrounding the 32-bp deletion in the CCR5 gene: 5′ primer, TCATTACACCTGCAGCTCTC; 3′ primer, TGGTGAAGATAAGCCTCAC. Wild-type CCR5 DNA results in a 197-bp product, but the Δ32 allele results in a 165-bp product. The genotype was determined by visual examination of the PCR product and of a known heterozygote used as a control.

The CCR5Δ32 allele was not found in the nonwhite patients, but it was found in 5 of the 9 white patients (Figure); overall allele frequency for white patients was 27.8%. Among the 5 who were heterozygous for the CCR5Δ32 allele, 1 died, 1 remained in the intensive care unit for >1 month, and 3 were discharged.

**Figure:**
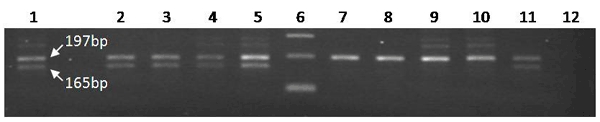
Amplification of the chemokine receptor 5 (CCR5) Δ32 locus in white patients. Lane 1, heterozygous positive control; lanes 2–5 and 7–11, patient samples; lane 6, 100-bp ladder; lane 12, negative control. CCR5Δ32 heterozygosity is observed in samples 2, 3, 4, 5, and 11.

## Conclusions

The outbreak of pandemic (H1N1) 2009 infection in Canada affected primarily young women; a preponderance were nonwhite and they had no major concurrent conditions. Risk factors identified included a history of lung disease or smoking, obesity, hypertension, and diabetes. The frequency of CCR5Δ32 heterozygosity among white populations has been reported to range from 10% to 15% (*12*,*13*); we found CCR5Δ32 heterozygosity at a higher than expected frequency (55.5%) among white patients with critical illness caused by pandemic (H1N1) 2009. Although deficiency of the receptor protects against acquisition of HIV, evidence is accumulating to suggest it plays a role in severity of illness caused by flavivirus infections (*7*,*8*). In animal models of influenza, CCR5 plays a role in directing CD8+ T cells to the site of infection, and its absence is associated with increased mortality rates (*9*,*10*); however, to our knowledge a similar association in humans has not yet been reported. Our observation suggests that CCR5Δ32 is 1 of the factors associated with increased severity of illness among white patients with pandemic (H1N1) 2009. Identifying genetic factors associated with greater risk for illness severity will help explain the unique pathogenesis displayed in the pandemic (H1N1) 2009 outbreak and may have public health implications. Further studies are required to illuminate the role of CCR5 in delivery of immune cells to the site of influenza infection.
